# Treatment of sirolimus in the pathological femoral fracture related to blue rubber bleb nevus syndrome: A case report

**DOI:** 10.1097/MD.0000000000029679

**Published:** 2022-07-29

**Authors:** Yu-Jui Chang, Jih-Yang Ko, Jiunn-Ming Sheen, Ka-Kit Siu

**Affiliations:** a Department of Orthopaedic Surgery, Kaohsiung Chang Gung Memorial Hospital and Chang Gung University College of Medicine, Kaohsiung, Taiwan; b Department of Medical Research, Kaohsiung Chang Gung Memorial Hospital and Chang Gung University College of Medicine, Kaohsiung, Taiwan; c Department of Pediatrics, Kaohsiung Chang Gung Memorial Hospital and Chang Gung University College of Medicine, Kaohsiung, Taiwan; d Department of Orthopaedic Surgery, Park One International Hospital, Kaohsiung, Taiwan.

**Keywords:** blue rubber bleb nevus syndrome, sirolimus

## Abstract

**Rationale:**

Blue rubber bleb nevus syndrome (BRBNS) is a rare condition with characteristic vascular malformations of the skin, most frequently lesions of the gastrointestinal tract and central nervous system, and less often, the musculoskeletal system. We report a 5-year case of BRBNS complicated with pathological femoral fracture that was successfully treated with sirolimus.

**Patient concerns:**

We report the case of a 1-week-old girl with a diagnosis of BRBNS who had multiple venous malformations over her body. She also presented with right lower-limb swelling and complicated with a pathological femoral fracture.

**Diagnoses:**

BRBNS with the complication of pathological femoral fracture.

**Interventions:**

Treatment with low-dose sirolimus as an antiangiogenic agent was administered, combined with hip spica protection.

**Outcomes:**

The vascular lesion was reduced after about 6 months and the fracture site had healed around 2.5 years after initiation of sirolimus therapy. There were no drug adverse effects at the 5-year follow-up point. The patient showed excellent spirit and no obvious sequelae were found.

**Lessons:**

To the best of our knowledge, this is the first report of the successful use of sirolimus in a patient with a pathological femoral fracture related to BRBNS complications.

## 1. Introduction

Blue rubber bleb nevus syndrome (BRBNS) is a rare vascular malformation featuring multiple organ venous malformations, including predominantly those of the skin, subcutaneous tissues, viscera, and gastrointestinal tract,^[1–4]^ less often the central nervous system, and very rarely the musculoskeletal system.^[5,6]^ Pathological fractures in infants are attributed to a variety of causes but are rare during the neonatal period.^[7]^ Accurate and careful analysis of the patient’s history, physical examination, and interpretation of radiographic and pathologic findings are crucial in order to deliver appropriate care. Sirolimus, an agent with an antiangiogenic property, can inhibit the production of vascular endothelial growth factor and the associated activity of vascular endothelial cells^[8, 9]^ and has been shown to be effective for the treatment of BRBNS.^[10–12]^

We report here a case with a right femoral pathological fracture as a clinical manifestation of BRBNS. We used a low dose of sirolimus as the treatment. After a 5-year follow-up period, the vascular lesion of the soft tissue and bone in the patient achieved remission and no obvious adverse drug effects were observed.

## 2. Case report

**Figure 1.**
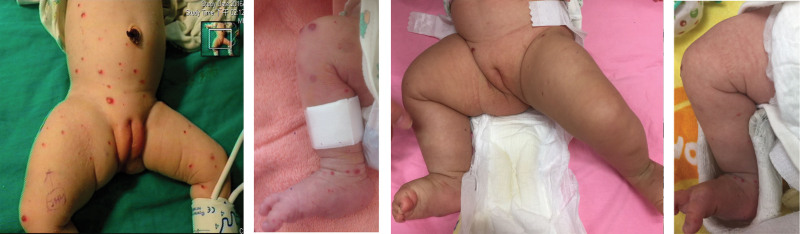
Representative images of skin lesion. Multiple vascular malformation characteristic with deep-blue, soft, rubbery blebs were found on the trunk and both lower limbs (A, B); regressed skin lesion after half year of sirolimus treatment (C, D).

**Figure 2.**
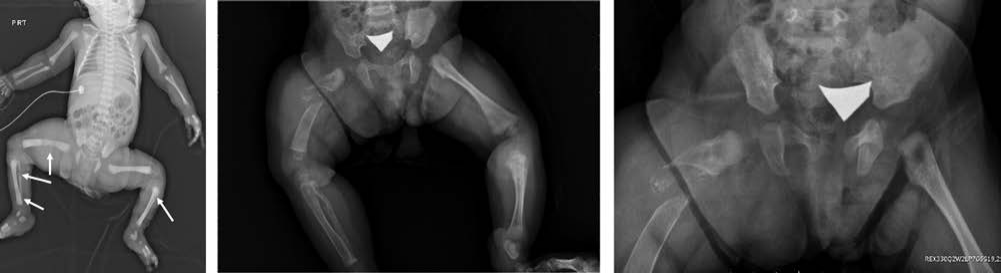
Multiple osteolytic lesions over both lower limbs (white arrows) (A) and evolved to the pathological femoral fracture after 3 mo (B, C).

**Figure 3.**
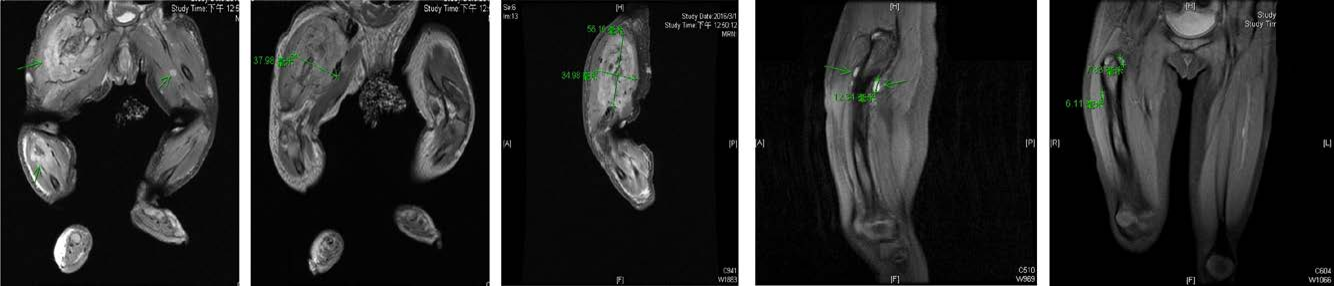
Musculoskeletal MRI showed multiple skin and soft tissue nodules throughout the lower trunk and both lower extremities with bone involvement. MRI = magnetic resonance imaging.

**Figure 4.**
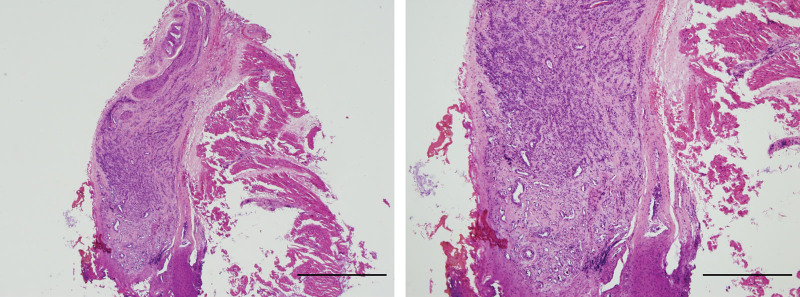
Histologic examination of the right thigh lesion showed aggregation of malformed capillaries with dilated vascular spaces in the skeletal muscle bundles. No definite malignancy was seen.

**Figure 5.**
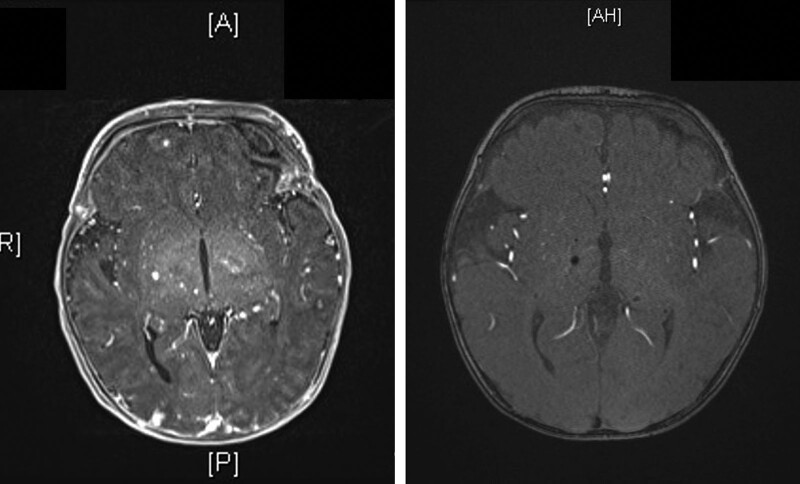
Brain MRI after 2 wk of birth showed bilateral cerebrum and cerebellum cavernomatosis (A) and regressed after 6 mo treatment of sirolimus (B). MRI = magnetic resonance imaging.

A 1-week-old girl, born to a 35-year-old mother at a gestational age of 40 + 3 weeks, was appropriate for the gestational age. After birth, multiple elevated, centrally-depressed, nonblanching deep-blue skin lesions were found over the patient’s head, trunk, and limbs, while the oral mucosa and vagina region were also involved (Fig. 1). The patient had no significant abnormal family history. On physical examination, obvious right thigh swelling with a deformity was found. The remainder of the physical examination was unremarkable. Plain film revealed osteolytic lesions with a periosteal reaction in the right femur and ipsilateral tibia and fibula (Fig. 2). Magnetic resonance imaging (MRI) of both lower limbs revealed multiple skin and soft tissue nodules throughout the lower trunk and both lower extremities, with bone involvement (Fig. 3). Laboratory findings showed mild macrocytic anemia and were as follows: hemoglobin, 13.1 g/dL (normal range, 14.2–17.2 g/dL), and mean corpuscular volume, 117.3 fL (normal range, 103.9–110.1fL; Table 1). A biopsy of the right thigh was performed, and pathologic result revealed aggregation of malformed capillaries with dilated vascular spaces in the skeletal muscle bundles (Fig. 4); no definite malignancy was observed. Otherwise, brain MRI showed bilateral cerebrum and cerebellum cavernomatosis (Fig. 5A).

**Table 1:** Analysis of initial basic laboratory data of patient.

**Parameter**	**Value**
WBC	9000/μL
RBC	3.36 million/μL
Hb	13.1 g/dL
Hematocrit	39.4%
MCV	117.3 fL
MCH	39.0 pg/cell
MCHC	33.2 g Hb/dL
RDW-SD	81.4 fL
Platelets	232,000/μL
INR	1.44
Prothrombin time	14.5 s
Activated partial thromboplastin time	43.7 s

**Figure 6.**
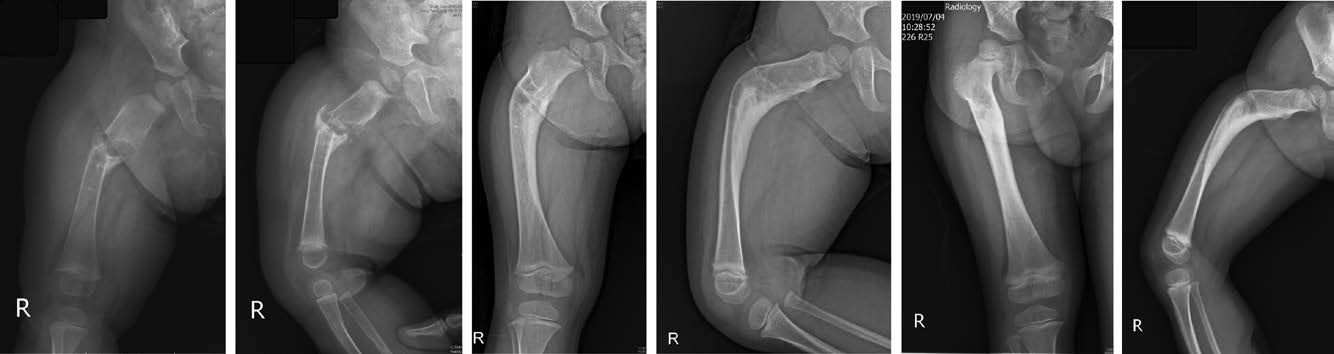
Right femur osteolytic lesion regressed and fracture site showed callus formation until 12 mo after the treatment of sirolimus (A, B); more callus formation noted after 2.5 yr follow-up (C, D); bone consolidation achieved after 3 yr follow-up (E, F).

**Figure 7.**
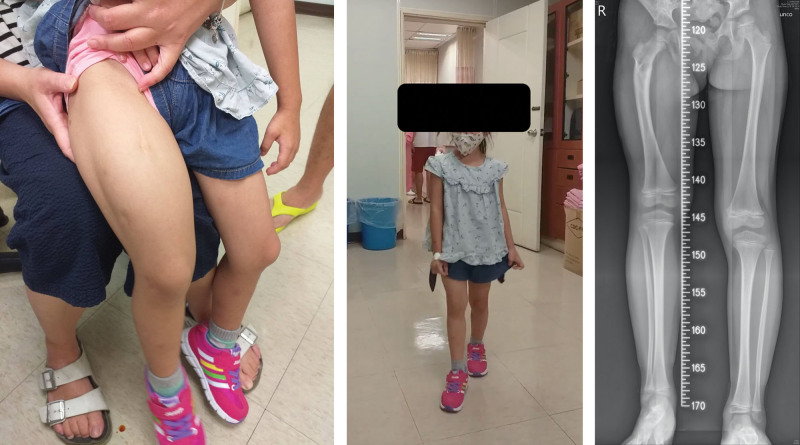
Nearly normal appearance of limbs on this patient after 5 yr follow-up (A, B); about 3-cm leg discrepancy is noted but the gait stable and no impact on her daily life (B, C).

Multidisciplinary experts, including a pathologist, hematologist, dermatologist, and pediatric geneticist, were consulted, and after discussion, BRBNS was diagnosed on the basis of the clinical, radiographic, and histologic findings. Then sirolimus (0.8 mg/m^2^) oral form was administered from the patient age of 1 month. A pavlik harness was fitted due to an impending right femur fracture initially, but after 3 months, a right femur pathologic fracture occurred, so it was then changed to a hip spica with leg protection extending to below the knee area. After 6 months of sirolimus therapy, the right thigh swelling subsided, and the follow-up brain MRI revealed regression of the cavernomatosis (Fig. 5B). Furthermore, the right femur fracture osteolytic lesion with nonunion regressed, and fracture-site healing with callus formation was noted at 12 months following initiation of sirolimus treatment (Fig. 6A, B), and more callus formation was noted after 2.5-year follow-up (Fig. 6C, D), then completed bone consolidation with remodeling after 3 years (Fig. 6E, F). After a 5-year follow-up period, there was a 3-cm leg discrepancy upon the latest examination, but the patient’s gait was stable and this had no obvious impact on her daily life (Fig. 7).

## 3. Discussion

Blue rubber bleb nevus syndrome (BRBNS) is a rare congenital vascular malformation involving more often in the skin and the GI tract and less often has skeletal involvement according to a literature review.^[3,5]^ The clinical manifestations of BRBNS are variable and depend on the involved organs.^[1,2]^ The prognosis depends on which organs are involved and the extent of involvement. However, BRBNS is an important condition because it can cause severe complications such as fatal bleeding.^[1,6,13]^ In our case, laboratory data revealed mild anemia and no blood transfusion was needed, while the most dominant presentations were vascular malformation of the skin and multiple osteolytic bone lesions.

The exact etiology of BRBNS remains unknown. Some research showed it to be of an autosomal dominant inheritance pattern with a locus found on chromosome 9p, while most cases are sporadic.^[14,15]^ Recently, *TEK/TIE2* mutations leading to this syndrome have been reported.^[16–18]^ The patient in our study had no significant family history of BRBNS. She did not receive genetic testing due to poor economic status and our limited detection method.

Pathological fractures in newborns may occur due to benign and malignant conditions.^[19]^ To the best of our knowledge, this was the first case of a pathological fracture in an infant due to BRBNS that showed a response to sirolimus, with no obvious complications noted after a 5-year follow-up period. Multidisciplinary approaches, including a thorough history review, physical examination, imaging studies, pathological analysis, and discussion with various specialists, including a dermatologist, hematologist, pediatric geneticist, pediatric orthopedist, and pathologist, are crucial to making a correct diagnosis and developing a treatment plan.

**Figure 8.**
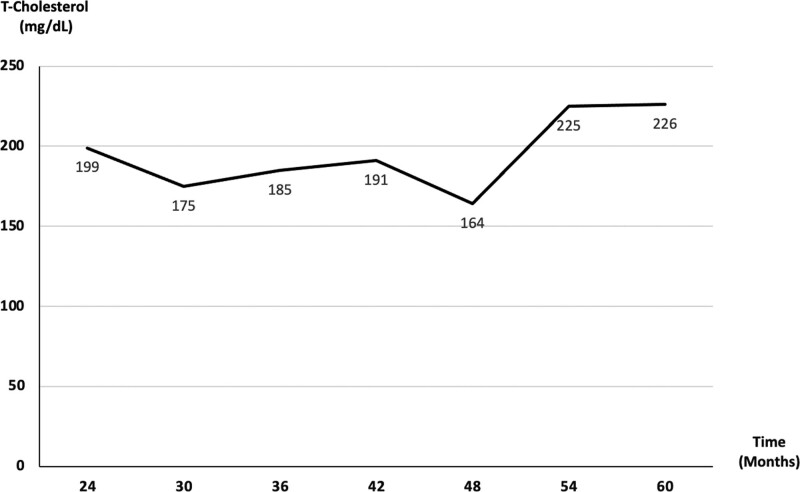
Follow-up total cholesterol level after sirolimus treatment.

Based on a review of the literature, there is no standard or curative therapy available for BRBNS.^[5]^ Surgical intervention is a curative option and could achieve successful treatment and generally in the clinical situation such as gastrointestinal lesions.^[1,6]^ Traditional medication treatment modalities include corticosteroids, interferon-α, which are antiangiogenic agents and could reduce the growth and proliferation of abnormal vascular endothelial cells. However, some research showed the patients did not respond to these agents.^[20,21]^ The mammalian target of rapamycin pathway regulates complex cellular processes including angiogenesis^[22]^ and is associated with tumorigenesis.^[23]^ Sirolimus is an mammalian target of rapamycin inhibitor and appears to be effective and safe in patients with vascular anomalies^[24]^ and has gained increasing popularity in the treatment of neoplastic conditions. Furthermore, it has shown some success in the treatment of BRBNS.^[10–13,25]^ However, the dose and the period of treatment of sirolimus for BRBNS are still controversial. Yuksekkaya et al^[10]^ adopted low-dose sirolimus (0.05–0.1 mg/kg) and the targeted blood level was between 1 and 5 ng/mL for an 8-year-old BRBNS girl. Successful clinical outcome was reported after around 2-year follow-up. Salloum et al^[12]^ also reported 4 cases of successful treatment with a dose of sirolimus ranging from 10 to 13 ng/mL. We adopted the low-dose sirolimus with the target blood level ranging from 4.5 to 12 ng/mL and also yielded acceptable outcome. However, sirolimus has potential adverse effects on renal function, bone marrow, and cholesterol metabolism. With regards to our case, there were no adverse drug effects or complications noted after sirolimus treatment for a 5-year period, with the exception of mild hypercholesterolemia, and we regularly monitor the cholesterol level (Fig. 8). There is no need of medical therapy for this currently.

## 4. Conclusions

BRBNS with a pathological fracture of the lower limb in an infant is a rare condition. Sirolimus treatment and orthosis protection were administered, and successful results were obtained after a 5-year follow-up period in the present case. Further studies are needed to evaluate the long-term effectiveness of sirolimus.

## Author contributions

Conceptualization: Jih-Yang Ko, Yu-Jui Chang

Data curation: Jih-Yang Ko, Yu-Jui Chang

Supervision: Ka-Kit Siu, Jiunn-Ming Sheen

Writing – original draft: Yu-Jui Chang

Writing – review & editing: Jih-Yang Ko, Jiunn-Ming Sheen, Yu-Jui Chang
